# Transduction of E2F-1 TAT fusion proteins represses expression of hTERT in primary ductal breast carcinoma cell lines

**DOI:** 10.1186/1476-4598-7-28

**Published:** 2008-03-26

**Authors:** Kimberly A Elliott, Lee F Rickords, J Marcelete Labrum

**Affiliations:** 1 Center for Integrated BioSystems, ADVS Department, Utah State University, Logan, UT 84322-4815, USA

## Abstract

**Background:**

Telomerase expression is detectable in 81–95% of breast carcinomas and may serve as a therapeutic target. The objective of this study was to investigate repression of telomerase activity in primary ductal breast cancer cells through transcriptional regulation of the catalytic subunit hTERT. We hypothesized that inhibition of telomerase expression could be achieved via Tat mediated protein transduction of the repressor protein E2F-1.

**Methods:**

Protein purification techniques were refined to yield biologically active Tat fusion proteins (TFPs) capable of transducing the breast cancer cell lines HCC1937 and HCC1599. Cell lines were treated with wildtype E2F-1 (E2F-1/TatHA), mutant E2F-1 (E132/TatHA) and a control Tat peptide (TatHA) for 24 hours. Total RNA was isolated from treated cells, reverse transcribed and fold changes in gene expression for hTERT determined via real-time RT-qPCR.

**Results:**

Significant repression of the catalytic subunit of telomerase (hTERT) was present in both HCC1937 and HCC1599 cells following treatment with E2F-1/TatHA. In HCC1937 cells, hTERT was repressed 3.5-fold by E2F-1/TatHA in comparison to E132/TatHA (p < 0.0012) and the TatHA peptide controls (p < 0.0024). In HCC1599 cells, hTERT was also repressed with E2F-1/TatHA treatment by 4.0-fold when compared to the E132/TatHA control (p < 0.0001). A slightly lower hTERT repression of 3.3-fold was observed with E2F-1/TatHA in the HCC1599 cells when compared to the TatHA control (p < 0.0001).

**Conclusion:**

These results suggest that transduction of E2F-1/TatHA fusion proteins in vitro is an effective repressor of hTERT expression in the primary ductal breast cancer cell lines HCC1937 and HCC1599.

## Background

Telomerase activity is detectable in 80–90% of malignancies and is absent in most normal somatic cells [1]. Breast cancer has been identified as an important target for developing telomerase inhibitors. Importantly, fine-needle aspirations of malignant breast tumors revealed that 81% were positive for telomerase [2], and Hiyama et al. (2000) also reported that 95% of advanced stage breast cancers express telomerase [3]. Due to the majority of tumor cells expressing telomerase, this protein is being evaluated as a tumor marker for breast cancer and other solid malignancies [4].

Telomerase, a ribonucleoprotein, synthesizes tandem repeats of the DNA sequence TTAGGG at the terminal ends of chromosomes permitting continuous genomic replication and cell division. Telomerase includes an RNA component (hTR) that serves as the template for telomeric DNA and a protein catalytic subunit (hTERT) with reverse transcriptase activity [5]. The activity of telomerase is regulated by the transcription of hTERT, and the cloning and characterization of the hTERT-promoter has permitted examination of elements controlling transcriptional activation and repression of hTERT [6].

To impact expression of hTERT in breast cancer cells, our research will utilize the E2F-1 transcription factor. The E2F family of transcription factors mediates cell cycle progression, and they are released upon phosphorylation of Retinoblastoma (Rb) family proteins [7-9]. While most of these factors are associated with inducing gene expression, E2F-1 has been identified as a transcriptional repressor of the hTERT gene, specifically binding to two separate sites (-174 bp and -98 bp) of the proximal hTERT promoter [7]. In addition, a study that examined the apoptotic function of E2F-1 found that cell death generated by ectopic expression of E2F-1 was independent of the p53 regulatory pathway [8]. Therefore, even in cells with intact p53 suppressor gene function, overexpression of E2F-1 should inhibit transcription of the hTERT gene and potentially induce apoptosis. Previous studies utilizing adenoviral-mediated transfection of E2F-1 genes induced apoptosis of melanoma cells [10], and an additional study revealed that E2F-1 suppressed cell growth while decreasing telomerase activity in the Tu-167 SCCHN cell line [11]. Adenoviral-mediated overexpression of E2F-1 also induced apoptosis in human breast and ovarian carcinoma cell lines independently of p53 [12]. We anticipate that telomerase repression via E2F-1 protein therapy could induce apoptotic activity and/or cell senescence in cancer cells more effectively than the viral-based studies described above.

Tat-mediated protein transduction was utilized in our research to effectively target cancer cells. Transduction occurs in a receptor- and transporter-independent fashion, targets the lipid bilayer, and also crosses the blood-brain barrier [13]. Tat has been reported to transduce 100% of mammalian cells, and studies also suggest the transcriptional effects of Tat-mediated protein transduction are reversible and have no detrimental effects because clearance of transduced proteins is dependent on the half-life of the protein [13-15]. Becker-Hapak et al. [14] also reported that Tat-mediated transduction is concentration dependent, reaches maximum intracellular concentration in less than 5 minutes, and nearly equal intracellular concentrations of the fusion proteins have been detected between all transduced cells.

Our research investigated primary infiltrating ductal carcinoma cells, both with and without a BRCA1 gene mutation. In 2001, published results by Ho et al. [16] determined that E2F-1 and E2F-4 were decreased in primary breast carcinomas, and 70% of the tumors revealed decreased expression of E2F-1. Additionally, the metastatic nodal tissue examined indicated that 100% of the tissue samples had significantly low levels of E2F-1 as compared to normal breast tissue. Ho et al. also suggested that E2Fs act as tumor suppressors in breast cancer and their down-regulation may be important in the development of metastases [16]. It is important to determine the repressive effects of E2F-1 on hTERT transcription in tumor cells with and without the normal BRCA1 protein to determine any variance between BRCA1 status and inhibition of telomerase. Recently, Wang et al. [17] determined that the BRCA1 promoter was transactivated in a dose-dependent manner by E2F-1 and served as a target for E2F-dependent transcriptional regulation. Other studies have also demonstrated that BRCA1 is a potent inducer of apoptosis by binding directly to p53 and stimulating transcriptional activation of pro-apoptotic genes such as Bax [18,19]. Therefore, telomerase inhibition in the primary infiltrating ductal carcinoma cell line with a BRCA1 gene mutation, HCC1937, will be compared to a breast cancer cell line without detectible BRCA1 gene mutations, HCC1599. Aside from variance in BRCA1 status, both HCC1937 and HCC1599 cell lines carry similar mutations in p53, Her2/neu and ER/PR receptors [20].

Here we report the first results of the repressive effects of E2F-1 on telomerase activity using Tat-mediated transduction. These studies validated our proposed hypothesis that telomerase activity can be repressed in infiltrating ductal carcinoma cells via transcriptional regulation of hTERT utilizing protein transduction techniques with E2F-1 Tat fusion proteins.

## Results

**Figure 1:**
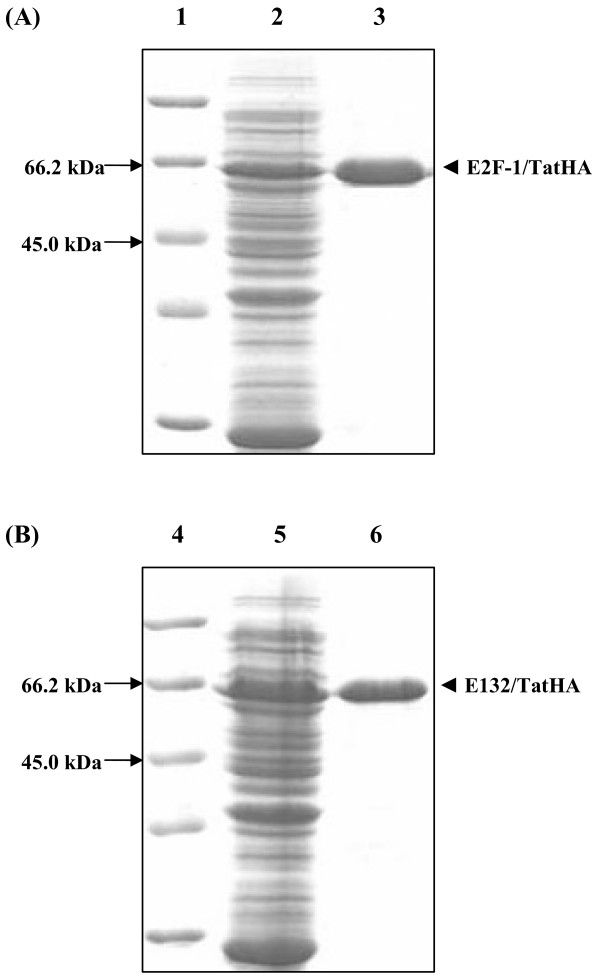
**SDS-PAGE gels of recombinant E2F-1/TatHA and E132/TatHA proteins**. SDS-PAGE gels of recombinant E2F-1/TatHA and E132/TatHA proteins. (A) SDS-PAGE gel of E2F-1/TatHA purified protein. *Lane 1*, Protein molecular weight marker. *Lane 2*, E2F-1/TatHA expressing culture. *Lane 3*, FPLC purified E2F-1/TatHA protein (60 kDa). (B) SDS-PAGE gel of E132/TatHA purified protein. *Lane 4*, Protein molecular weight marker. *Lane 5*, E132/TatHA expressing culture. *Lane 6*, FPLC purified E132/TatHA protein (60 kDa).

**Figure 2:**
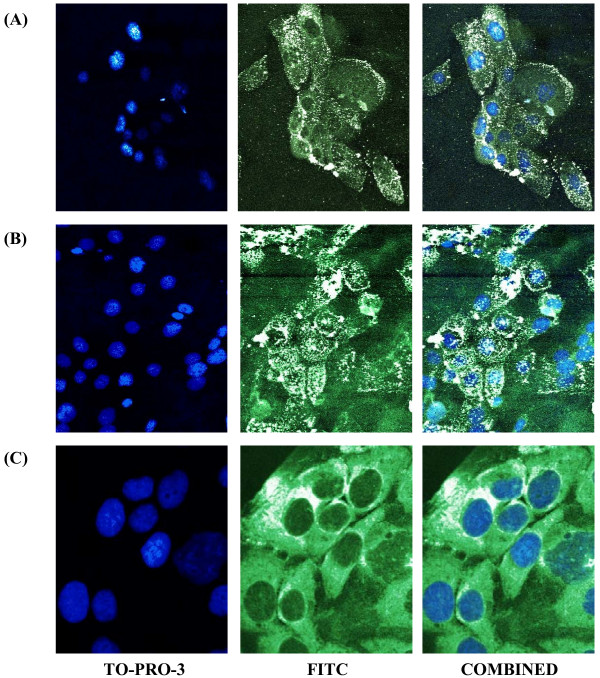
**Immunocytochemistry images of HCC1937 cells**. Immunocytochemistry images of HCC1937 cells transduced with Tat fusion proteins for 24 hours. (A) Cells treated with 2 μM E2F-1/TatHA. (B) Cells treated with 2 μM E132/TatHA. (C) Cells treated with 2 μM TatHA control peptide. Images were captured using the 40× objective on the Bio-Rad MRC 1024 Laser Scanning Confocal Microscope.

We were able to successfully produce E2F-1/Tat fusion peptides utilizing both wildtype and mutant E2F-1 expression vectors to investigate their transcriptional effects on hTERT. Techniques were modified to produce both E2F-1/TatHA and E132/TatHA recombinant proteins (see Figure 1) and recombinant protein production was verified via western blotting. Protein transduction was determined in each cell line via immunocytochemistry, and the results supported effective transduction of E2F-1/TatHA, E132/TatHA and TatHA into all cell types. The TFPs were detected throughout the cell cytoplasm with perinuclear localization and detectable nuclear accumulation (Figure 2). The series of immunocytochemistry experiments ranging from 1 hour to 24 hours incubation with TFPs all revealed successful transduction of recombinant proteins into greater than 95% of carcinoma cells.

**Figure 3:**
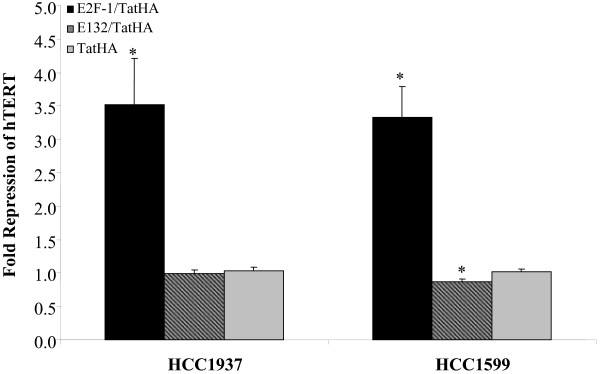
**Fold repression of hTERT gene expression in breast cancer cell lines- TatHA control**. Fold changes in hTERT gene expression compared to TatHA control in breast cancer cell lines. HCC1937 and HCC1599 cells were treated for 24 hours with 2 μM Tat fusion proteins. RNA was isolated and RT-qPCR performed as described under Materials and Methods. The asterisks (*) indicate statistically significant fold changes in gene expression. A significant 3.5-fold repression of hTERT gene expression was observed in HCC1937 cells treated with E2F-1/TatHA proteins in comparison to treatment with TatHA control proteins (p < 0.0024). Repression of hTERT in HCC1937 cells treated with E132/TatHA proteins versus the TatHA control proteins was not statistically significant (p < 0.6484). A 3.3-fold repression of hTERT was observed in HCC1599 cells treated with E2F-1/TatHA proteins versus the TatHA control protein (p < 0.0001). hTERT gene expression was slightly increased (1.15-fold increase) in HCC1599 cells treated with E132/TatHA proteins versus the TatHA control proteins (p < 0.0102). The results are means +/- SEM of three separate experiments (HCC1937, n = 17; HCC1599, n = 18).

Our experiments were only performed with freshly dialyzed proteins to avoid protein precipitation and to ensure biological activity of proteins. The proteins were added to cells for 24 hours to assess differences in hTERT expression between the wildtype E2F1/TatHA protein, mutant E2F-1 (E132/TatHA) and the TatHA control protein. Following RNA isolation and real-time RT-qPCR, results revealed significant repression of hTERT in both HCC1937 and HCC1599 breast cancer cells. In HCC1937 cells, E2F-1/TatHA greatly repressed hTERT by 3.5-fold (p < 0.0024) (Figure 3) in comparison to the TatHA control proteins. Furthermore, the cells treated with E132/TatHA did not reveal effective repression of hTERT in comparison to TatHA (p < 0.6484).

In HCC1599 cells, a significant 3.3-fold repression of hTERT gene expression was observed with E2F-1/TatHA protein treatment versus the TatHA control protein (p < 0.0001) following 24 hours of treatment (Figure 3). Also, a slightly increased expression of hTERT (1.15-fold increase) was observed in TatHA treated HCC1599 cells versus the E132/TatHA protein, but this difference was only marginally statistically significant (p < 0.0102) (Figure 3). Further testing will be performed to assess differences between E132/TatHA and TatHA control proteins in larger in vitro test groups.

Minimal differences in hTERT expression were detected in HCC1937 and HCC1599 cells based on the BRCA1 status. As previously mentioned, the HCC1937 cell line has a homozygous BRCA1 mutation (5382C) in addition to mutations in p53, Her2/neu and ER/PR receptors. In contrast, the HCC1599 cell line expresses the wildtype BRCA1 tumor suppressor gene but also contains mutations in p53, Her2/neu and ER/PR receptors. A slightly higher repression of hTERT was observed in HCC1599 cells (4.0-fold) versus HCC1937 (3.5-fold) when E2F-1/TatHA was compared to the E132/TatHA control, but this slight difference in hTERT repression is most likely not attributed to the difference in BRCA1 status.

## Discussion

The results from this study suggest that E2F-1/TatHA is a moderately effective repressor of hTERT. In comparison to E132/TatHA and TatHA controls, hTERT was repressed by 3.3 to 4.0-fold in HCC1937 and HCC1599 breast carcinoma cells. The efficient transduction of the E2F-1/TatHA peptides into breast cancer cells combined with their transcriptional effects on hTERT expression suggests an alternate route to inhibit telomerase expression in breast cancer cells. The extent of hTERT repression in our study is comparable to the results observed by Crowe et al.'s study (2001) which suggests that our alternative route of introducing the E2F-1 transcription factor into cancer cells does not result in greater repression of hTERT than liposome-mediated transfection of E2F-1 [7]. However, the introduction of biologically active E2F-1/TatHA proteins has potential benefits. Treatment with our wildtype and mutant TFPs results in reversible repression of hTERT which could serve as a modality to augment the effects of other chemotherapeutic or apoptotic agents. This particular study did not examine the direct effects on apoptosis, but continued treatment with E2F-1/TatHA proteins could serve to effectively repress hTERT leading to long-term telomerase repression.

To interpret the results of our hTERT repression studies, it is important to review the expected effects of E2F-1/TatHA treatment of cells versus E132/TatHA and control peptide TatHA. Crowe et al. determined the normal E2F-1 expression vector binds to two sites of the hTERT promoter and represses its transcription by approximately 4-fold in their study [7]. The negative control, mutant E2F-1, referred to as E132/TatHA in our research, has a point mutation in the DNA-binding domain and cannot bind to the E2F-1 binding sites on the hTERT promoter and exhibited no repressive effects on hTERT [7]. We expected to observe similar results in our study, and since Tat-mediated protein transduction is an effective method of delivering biologically active proteins into cells, we hoped to achieve greater than 4.0-fold repression of hTERT in our study. However, as demonstrated by our data following 24 hours of treatment with E2F-1/TatHA, only a maximum of 4-fold hTERT repression was observed in HCC1599 cells.

The characterization of E2F-1 as a transcriptional repressor is a controversial role for this factor because it has traditionally been denoted as a transactivator of various genes. Hsieh et al. [21] reported that transcriptional repression rather than the transactivation function of E2F-1 is involved in the induction of apoptosis. Zhang et al. [19] provided the first direct evidence that telomerase is required for maintaining the vitality of both human tumor and immortal cells. Zhang et al. also reported that cell death in immortal cells was telomere-length dependent, and upon inhibiting telomerase in cells with short telomeres, the resulting damage to the chromosomes triggered apoptotic cell death [19]. Interestingly, Yamasaki et al., [22] determined that E2F-1 null mutant mice developed a variety of malignant tumors, and Field et al. [23] indicated that E2F-1 functions in mice to promote apoptosis and suppress cell proliferation. Tumor formation in the absence of E2F-1 was also suggested by Crowe et al. [7]. Their study utilized E2F-1 expression vectors and liposomal transfection into SCC25 cells resulting in transcriptional repression of hTERT via consensus binding in the hTERT- promoter at putative E2F-1 sites [7]. Importantly, Crowe et al. reported that transfection of the E2F-1 constructs reduced endogenous hTERT mRNA levels by 4-fold, and the reduced hTERT mRNA levels were accompanied by decreasing telomerase activity [7]. The study by Crowe et al. was one of the first to correlate the novel transcriptional repressive effects of E2F-1 on the hTERT gene promoter while monitoring subsequent telomerase activity [7]. However, the repressive effects of E2F-1 on hTERT must be further examined in future studies. The liposome-mediated transfection of E2F-1 via the Lipofectamine™ kit (Invitrogen Life Technologies, Carlsbad, CA) as described in Crowe et al. does not guarantee transfection of 100% of the cancer cells [7], nor do any of the adenoviral-mediated studies previously mentioned [10-12]. Therefore, to determine if E2F-1 can completely repress hTERT transcription, the E2F-1 protein must be effectively transduced into 100% of the cancer cells. Our research suggested greater than 95% transduction rates with E2F-1 TFPs, but maximum hTERT repression was only 4-fold with our alternative mode of transduction, which is comparable to results published by Crowe et al [7]. Complete repression of hTERT was not observed in our study using TFPs suggesting that a combination of transcriptional repressors may be required when using Tat-mediated transduction to completely inhibit expression of hTERT. It must be noted, that to date, complete gene repression has only been demonstrated utilizing gene knockout protocols and models. There are, however, significant advantages of using our method of transducing E2F-1 TFPs over transfection protocols. One is the simplicity of being able to use a TAT-peptide to transport any denatured protein inside the cell. Another is that it alleviates the deleterious effects associated with viral based DNA transfection procedures and does not result in permanent genetic manipulation. In addition, therapeutic treatment with these TFPs is completely reversible, permitting transient repression of target genes.

## Conclusion

Our lab refined an efficient protocol to produce and purify E2F-1/TatHA and E132/TatHA proteins by using strong denaturants, FPLC and dialysis. The biologically active E2F-1/TatHA, E132/TatHA and TatHA proteins effectively transduced greater than 95% of cell lines tested as detected via immunocytochemistry. Following treatment of cell lines with E2F-1/TatHA, E132/TatHA and TatHA proteins for 24 hours, our data revealed significant repression of hTERT with E2F-1/TatHA treatment.

In HCC1937 cells, hTERT was repressed 3.5-fold by E2F-1/TatHA in comparison to E132/TatHA and the TatHA peptide controls. In HCC1599 cells, hTERT was also repressed with E2F-1/TatHA treatment by 4.0-fold when compared to the E132/TatHA control. A slightly lower hTERT repression of 3.3-fold was observed with E2F-1/TatHA in the HCC1599 cells when compared to the TatHA control. No differences were observed in cell lines with wildtype versus mutant BRCA1 genes. Overall, these results suggest that transduction of E2F-1/TatHA fusion proteins in vitro is a moderately effective repressor of hTERT expression in the primary ductal breast cancer cell lines HCC1937 and HCC1599. The hypothesized repression of telomerase activity in infiltrating ductal carcinoma cells via E2F-1 repressor proteins is supported by significant hTERT repression in both HCC1937 and HCC1599 cells.

The breast cancer cell lines utilized in our study represent non-metastatic primary ductal carcinoma cell lines. HCC1937 cells were derived from a TNM stage IIB, grade 3 breast tumor, and the HCC1599 cell line was started from a TNM stage IIIA, grade 3 primary ductal carcinoma [20]. Our research suggests that E2F-1/TatHA is an effective repressor of hTERT in primary ductal carcinoma cells that are actively expressing telomerase. Although we have not fully investigated metastatic breast carcinoma cell lines, preliminary studies (results not shown) with MDA-MB-231 and MDA-MB-435S cells, both of which are derived from pleural effusions from metastatic breast cancer (TNM stage IV) [17], did not reveal statistically significant repression of hTERT. Therefore, the E2F-1/TatHA proteins investigated in this study would be potential adjuvants to therapy in nonmetastatic, primary breast tumors.

Collectively, these studies were undertaken to develop an effective technique to achieve complete transduction of cancer cells with fusion peptides. The hypothesized repression of telomerase activity in infiltrating ductal carcinoma cells via E2F-1 repressor proteins is supported by significant hTERT repression in both HCC1937 and HCC1599 cells. Future experiments will further investigate the transcriptional repressive effects of E2F-1 and its effects on cell-cycle progression and telomerase repression. To conclude, this therapy can initiate a sufficient biological effect to induce cell death in cancer cells but is expected to be safe enough to permit normal cells to recover from treatment without genetic alteration. Further research with E2F-1 TFPs will continue to explore their therapeutic potentials and benefits in the field of breast cancer research.

## Methods

### Vector DNA

The vectors pCMV E2F-1 and pCMV E132 were obtained from Dr. Karen Vousden (Beatson Cancer Institute, UK) and the pTatHA vector was a kind gift from Dr. Steven Dowdy (University of Washington, St. Louis, MO).

### E2F-1/TatHA and E132/TatHA constructs

The wildtype E2F-1 gene was directly cloned into the pTatHA vector via NcoI and EcoRI restriction enzyme sites. The E132 vector (mutant E2F-1) contained an artificial EcoRI site in the DNA binding domain and could not be directly inserted into pTatHA's multiple cloning site (MCS). Therefore, the E132 gene was modified using 5' site-directed mutagenesis via PCR from plasmid pCMV-E132 using sense primer: 5'-gcgcgcaaccATGGCCTTGGCCGGG-3'and antisense primer: 5'-gcgcagcatgcGGATCCAGCCCTGTC-3' to generate artificial NcoI and SphI sites (underlined in primer sequences). The PCR reaction used 2 Units Vent Taq polymerase (New England Biolabs, Beverly, MA), 1× Thermapol buffer (New England Biolabs, Beverly, MA), 500 nM of sense and antisense primers and 5% DMSO. The PCR product was amplified under the following conditions: initial cycle 94°C- 3 minutes, 55°C- 1 minute, 72°C- 1 minute followed by 23 cycles of 94°C- 1 minute, annealing, 55°C- 1 minute, 72°C- 1 minute and a final extension of 72°C for 7 minutes. The pTatHA DNA was digested with restriction enzymes NcoI and SphI and purified using phenol/chloroform extraction. The E132 PCR product was digested with NcoI-SphI and subcloned into the NcoI-SphI sites of pTatHA to yield E132/TatHA using a Klenow-Kinase-Ligase (KKL) protocol [24].

### Recombinant protein production

E2F-1/TatHA and E132/TatHA plasmids were transformed into BLR(DE3)pLysS competent cells (Novagen, Madison, WI) and recombinant proteins produced via inoculating 2 ml 2YT broth with single transformed colonies. After incubation in shaking water bath (225 rpm) for 12–14 hours, 3 ml of fresh 2YT broth, 0.5 μg/ml ampicillin, and 0.4 mM isopropyl-beta-D-thiogalactopyranoside (IPTG) were added to cultures. Cultures were incubated an additional 6 hours to produce recombinant proteins via IPTG stimulation. Cultures were centrifuged at 6000 × g for 10 minutes and cell pellets resuspended in 100 μl of 20 mM Tris buffer (pH 8.0) followed by quantitation using a BCA™ Protein Assay Kit (Pierce Biotechnologies, Inc., Rockford, IL) with BSA standards. 2× Laemmli Sample Buffer was added to 50 μg of total cellular protein and samples were boiled for 3–5 minutes and immediately placed on ice [25]. Samples were then loaded onto 12% SDS-PAGE gels and run on a Mini-PROTEAN^® ^3 (Bio-Rad Laboratories, Hercules, CA) protein electrophoresis unit using the Laemmli buffer system. Prestained 1 kb protein ladders (Fermentas Life Technologies, Hanover, MD) were also run on each gel to determine protein size (refer to Figure 1).

### Western blotting

For Western blotting experiments, duplicate gels were run simultaneously- one gel for western blotting and a second gel for Coomassie blue staining. The gel designated for western blotting was transferred to nylon membranes using a Trans-Blot SD Semi-Dry Electrophoretic Transfer Cell (Bio-Rad Laboratories, Hercules, CA) according to manufacturer's instructions using 1× TBS-T transfer buffer. Recombinant protein production was verified using primary mouse monoclonal anti-HA antibodies (1:1,000 dilution) (CRP, Inc., Denver, PA) and secondary HRP-labeled goat anti-mouse antibodies (1:25,000 dilution) (KPL, Inc., Gaithersburg, MD). Membranes were blocked with TBS-T/5% milk solution for 1 hour, incubated with primary anti-HA antibody for 1 hour, washed 3 times for 10 minutes each, and incubated with secondary HRP antibody for 1 hour. Following additional washings (3 × 10 minutes), western blots were developed with SuperSignal West Pico Chemiluminescent Substrate (Pierce Biotechnology, Inc., Rockford, IL) according to manufacturer's instructions. Blots were exposed to X-ray film and developed.

### Large-scale recombinant protein production

Recombinant E2F-1/TatHA and E132/TatHA proteins were purified under denaturing conditions with affinity chromatography using a modified procedure from Amersham Pharmacia [26]. Single colonies from protein expressing cultures inoculated 200 ml of 2YT broth supplemented with ampicillin (50 μg/ml) and were incubated at 37°C while shaking at 225 rpm for 16–18 hours. An additional 300 ml of broth was added along with fresh ampicillin and IPTG (400 μM), and cultures incubated an additional 6 hours. Cultures were centrifuged for 10 minutes at 6000 × g, and the pellets frozen at -20°C. Cell pellets were thawed for 20 minutes at 37°C, and 25 ml of Buffer 1 (20 mM Tris™-HCl, pH 8.0) added to resuspend the cells with vigorous pipetting. Cells were sonicated on ice (3 × 15 sec) and centrifuged (10 min × 6000 g). Cell pellets containing the inclusion bodies were then resuspended in 25 ml Buffer 2 (2 M urea, 20 mM Tris-HCl, 0.5 M NaCl, 2% Triton™ X-100, pH 8.0). Samples were again sonicated on ice and centrifuged as described above. Pellets were resuspended in 25 ml Buffer 2 to wash the inclusion bodies and recentrifuged. A final wash of 25 ml of Buffer 1 was then performed, and following centrifugation, pellets were either used immediately or frozen at -20°C for a maximum of two weeks prior to use.

### Preparation of inclusion bodies for FPLC

Cell pellets were resuspended in 5–10 ml Buffer A1 (6 M guanidine-HCl, 0.5 M NaCl, 20 mM Tris-HCl, 20 mM imidazole, 5 mM β-mercaptoethanol, pH 8.0) by stirring at room temperature for 60 minutes to solubilize recombinant proteins. Samples were centrifuged at 20,000 × g for 15 minutes to pellet residual cell debris. Protein supernatant containing denatured, soluble proteins was removed from residual cell debris. Supernatant was further clarified by passage through a 0.45 μM filter (Millipore, Bellerica, IL) to further remove any particles.

### Isolation of recombinant proteins using affinity chromatography

HisTrap™ Ni^++ ^charged columns (Amersham Pharmacia, Piscataway, NJ) were equilibrated with 5 ml Buffer A1 and the proteins loaded at a flow rate of 0.5 ml/minute. Columns were washed with an additional 10 ml of Buffer A1 followed by a wash of 10 ml of Buffer A2 (6 M urea, 0.5 M NaCl, 20 mM Tris-HCl, 20 mM imidazole, 5 mM β-mercaptoethanol, pH 8.0). A linear gradient from 6 M to 0 M urea was then performed to remove the denaturant and start refolding the proteins on the HisTrap™ column by gradually replacing Buffer A2 with Buffer B1 (0.5 M NaCl, 20 mM Tris-HCl, 20 mM imidazole, 5 mM β-mercaptoethanol, pH 8.0). A total volume of 40 ml of Buffer B1 was used to perform the gradient wash. The purified, refolded proteins were then eluted with Buffer B2 (0.5 M NaCl, 20 mM Tris-HCl, 500 mM imidazole, 5 mM β-mercaptoethanol, pH 8.0) using a gradient of 0 mM to 500 mM imidazole in the buffer. Fractions containing the purified protein were analyzed by SDS-PAGE gel electrophoresis and quantified with the BCA™ Protein Assay Kit (Pierce Biotechnology, Inc., Rockford, IL).

### Dialysis of recombinant proteins into cell culture media

E2F-1/TatHA and E132/TatHA recombinant proteins were diluted with Buffer B2 to a concentration of 0.5 mg/ml to avoid protein precipitation cascades and injected into 12 ml Slide-A-Lyzer^® ^Dialysis Cassettes (Pierce Biotechnology, Inc., Rockford, IL) using 21-gauge, 1-inch beveled hypodermic needles. Proteins were dialyzed in 1× PBS for 2 hours at room temperature. The cassettes were placed in fresh 1× PBS for an additional 2 hours at room temperature and then dialyzed overnight in appropriate cell culture media for designed cell line experiments. Following dialysis, proteins were removed from cassettes and placed in sterile 50 ml conical centrifuge tubes and centrifuged at 9400 × g for 15 minutes to remove any protein precipitate. Protein supernatant was removed and the final concentrations of recombinant proteins determined using the Coomassie Plus™ Bradford Assay (Pierce Biotechnology, Inc., Rockford, IL) and SDS-PAGE gels using BSA protein standards. Contamination of proteins was minimized by passage through 0.2 μM filters (Millipore, Bellerica, IL). Purified protein was supplemented with 10% heat-inactivated FBS (Hyclone, Logan, UT) and 50 IU/ml penicillin/0.05 mg/ml streptomycin. Prior to testing on carcinoma cell lines, various μM concentrations were tested to determine optimal transduction at different time points using immunocytochemistry to monitor cellular uptake of E2F-1/TatHA and E132/TatHA.

### Synthesis of TatHA control protein

The TatHA control protein was artificially synthesized (amino acid sequence: YPYDVPDYAYGRKKRRQRRR) [27] on a Ranin Symphony/Multiplex Peptide Synthesizer (Center for Integrated BioSystems, Logan, UT). The TatHA peptide was reconstituted using Cellgro™ RNase free/DNase/Protease free dH_2_0 for cell culture (Mediatech Inc., Herndon, VA) and stored at -70°C at a working concentration of 1 mg/ml [28].

### Primary carcinoma cell culture

Primary infiltrating ductal breast carcinoma cell lines HCC1937 (ATCC number: CRL-2336) and HCC1599 (ATCC number: CRL-2331) were obtained from the American Type Culture Collection (Manassas, VA). The cells were grown in RPMI-1640 medium supplemented with 10% FBS, 50 IU/ml penicillin/0.05 mg/ml streptomycin, 10 mM HEPES, and 1 mM sodium pyruvate. Cells were grown in a 37°C humidified incubator with 5% CO_2_. Cell lines were grown in 25 cc^2 ^and 75 cc^2 ^tissue culture flasks and passaged every 2–4 days when 70–80% confluent. Cells were detached from flasks using 0.25% trypsin, 0.03% EDTA solution according to standard cell culture protocols [29].

### Immunocytochemisty

HCC1937 and HCC1599 cells were exposed to 2 μM E2F-1/TatHA, E132/TATHA, and TatHA fusion peptides for various time points: 1, 6, 12 and 24 hours. Cells were washed 3 times with 1× PBS, fixed for 30 minutes in 3.7% formaldehyde and permeabilized with 0.5% Triton X-100 in 1× PBS for 30 minutes. Cells were blocked for 30 minutes with 1% FBS and 0.1% Tween-20 in 1× PBS. Cells were incubated for 60 minutes with primary mouse HA antibodies (1:1000 dilution in 1× PBS), washed 3 times for 10 minutes each, and incubated for 60 minutes with a secondary FITC-labeled goat anti-mouse antibody (1:250 dilution in 1× PBS) (KPL, Inc., Gaithersburg, MD). Cells were then washed, and incubated for 15 minutes with 1 μM TO-PRO-3 iodide (Molecular Probes, Eugene, OR) to counterstain the cell nuclei. Cells were then mounted on slides using Prolong^® ^Antifade (Molecular Probes, Eugene, OR) and images captured with Bio-Rad MRC 1024 Laser Scanning Confocal Microscope (see Figure 2).

### Cell culture experiments with recombinant proteins

HCC1937 and HCC1599 cells were seeded at a density of 2 × 10^5 ^cells/well in CellStar^® ^6-well tissue culture plates. HCC1937 (adherent) cells were allowed 24 hours to adhere to plates before recombinant proteins were added. HCC1599 (suspension) cells were seeded directly into wells with TFPs. After 24 hours of co-incubation, RNA was isolated and analyzed via RT-qPCR as described below.

### RNA isolation, reverse transcription and quantitative PCR

Total RNA was isolated from cells using the Mini RNA Isolation II™ kit (Zymo Research, Orange, CA) using the following protocol. After treatment with TFPs, adherent cell lines were washed at least three times with 1× PBS and detached from tissue culture plates using 0.5 ml HyQ^®^tase™ (Hyclone, Logan, UT). Cells were added to 1.8 ml microcentrifuge tubes and pelleted by centrifugation at 14,000 rpm for 1 minute. Supernatant was decanted and excess fluid removed via micropipetting. A 600 μl volume of ZR RNA Buffer was added to cells, tubes vortexed 30 seconds, and sample transferred to a Zymo-Spin III column placed in a 2 ml collection tube. Zymo-Spin III column was centrifuged at 14,000 rpm for 1 minute. A 350 μl volume of RNA Wash Buffer was added to the Zymo-Spin III column and centrifuged as described above to wash column. A second wash was performed with 350 μl RNA Wash Buffer to further clean RNA. The spin column was then transferred to a sterile 1.8-ml microcentrifuge tube. A 50 μl volume of RNase-free water was added directly to the membrane of the Zymo-Spin III Column and column centrifuged for 15 seconds to elute RNA. The eluted RNA was quantified using a Nanodrop^® ^ND-1000 Spectrophotometer (Nanodrop Technologies, Wilmington, DE).

A two-step reverse transcriptase-quantitative polymerase chain reaction (RT-qPCR) was performed for all the experiments described in this study. A 200 ng quantity of RNA was treated with DNase (1 U/μg) (Fisher Scientific, Pittsburgh, PA) in a 10 μl digestion reaction volume including a final 1× concentration of RQ1 RNase-Free DNase Reaction Buffer (40 mM Tris-HCl, 10 mM MgSO_4_, 1 mM CaCl_2_, pH 8.0) (Fisher Scientific, Pittsburgh, PA). Samples were incubated at 37°C for 30 minutes followed by the addition of 1 μl of RQ1 DNase Stop Solution (20 mM EGTA, pH 8.0) (Fisher Scientific, Pittsburgh, PA) and samples incubated at 65°C for 10 minutes to inactivate the DNase. The 11 μl volume of DNase-digested RNA was used for the RT-PCR reaction.

### Production of cDNA from total RNA

The 11 μl volume containing the RNA was combined with 2 μl random hexadeoxynucleotides (0.5 μg/ul) (Fisher Scientific, Pittsburgh, PA) and a 4 μl volume of dNTP mix (10 mM per dNTP) (New England Biolabs, Beverly, MA). Tubes were heated for 5 minutes at 70°C to anneal primers to RNA, samples then placed on ice, and the following components added to the reaction tube to yield a 20 μl volume: 2 μl 10× RT Buffer (50 mM Tris-HCl, 75 mM KCl, 3 mM MgCl2, 10 mM DTT, pH 8.3) (New England Biolabs, Beverly, MA), 10 U of RNase Inhibitor (Fisher Scientific, Pittsburgh, PA), and 25 U M-MuLV Reverse Transcriptase (New England Biolabs, Beverly, MA). Reaction tubes were incubated at 42°C for 1 hour to generate cDNA, and the RT enzyme heat inactivated at 95°C for 5 minutes. A 0.5 μl volume containing 2.5 U of RNase H (New England Biolabs, Beverly, MA) was added to tubes and samples incubated at 37°C for 20 minutes to degrade RNA. RNase H was then heat inactivated at 95°C for 5 minutes. Each cDNA pool was diluted to 50 μl and stored at -20°C until analyzed via real-time qPCR reaction.

### Real-time quantitative PCR

Real-time qPCR was performed using the MyiQ™ Single Color Real-Time PCR Detection System (Bio-Rad, Hercules, CA). A 2 μl sample of each cDNA pool served as template for the qPCR reaction using IQ™ SYBR^® ^Green Supermix (Bio-Rad, Hercules, CA) and 300 nM of each primer: hTERT sense & antisense primers (5'-GGAGCAAGTTGCAAAGCATTG-3' & 5'-CCCACGACGTAGTCCATGTT-3') [30], B-actin sense & antisense primers (5'-CACTCTTCCAGCCTTCCTTCC-3'& 5'-CTGTGTTGGCGTACAGGTCT-3') [31]. All qPCR reactions were performed in at least triplicate for each sample using 96-well plates. The following cycling program was used for all primer sets. Initial denaturation step at 95°C for 3 minutes followed by 45 cycles at 94°C- 30 sec., 59.5°C- 30 sec., 72°C- 30 sec., and an 80°C- 15 sec. data acquisition step. A melt curve was performed for every reaction plate. Fold changes in gene expression were determined using the 2^-ΔΔCT^method [32]. The C_T _data were imported into Microsoft Excel. C_T _values for hTERT and B-actin were averaged for each sample, and ΔC_T _calculated (ΔC_T _= C_T, avg. hTERT _– C_T, avg. B-actin_). ΔΔC_T _was calculated in the following manner: ΔΔC_T _= Avg. ΔC_T, hTERT from E2F-1 or E132 TFP treatment _– ΔC_T, hTERT from TatHA control_. Mean fold change in gene expression was determined by the following calculation: 2^^-ΔΔCT^.

### Statistical analysis

Statistical significance of mean fold changes in gene expression was determined by Student's paired t-test using Microsoft Excel and Analyze-it™ (Analyze-it Software, Ltd., vsn 1.71). Results were considered significant if two-tailed P values were < 0.05.

## Abbreviations

BSA, bovine serum albumin; BRCA1, breast cancer susceptibility gene 1; DMSO, dimethyl sulfoxide; FPLC, fast performance liquid chromatography; FBS, fetal bovine serum; FITC, fluorescence isothiocyanate HA, Hemagluttin; HRP, horseradish peroxidase; IPTG, isopropyl-beta-D-thiogalactopyranoside; KKL, Klenow-Kinase-Ligase; MCS, multiple cloning sites; PBS, phosphate buffered saline; Rb, retinoblastoma; RT, reverse transcriptase; RT-qPCR, reverse transcriptase quantitative polymerase chain reaction; SDS-PAGE, sodium dodecyl sulfate-polyacrylamide gel electrophoresis; TFP(s), Tat fusion protein(s); Tat, transactivator of transcription; TatHa; transactivator of transcription with Hemagluttin-tag; TBS-T, Tris buffered saline-Tween 20.

## Competing interests

The author(s) declare that they have no competing interests.

## Authors' contributions

KE performed cloning experiments, developed the protein purification protocols, developed real-time qRT-PCR experiments and experimental design. KE also performed statistical analysis and drafted manuscript. LR was involved in experimental design, coordinated the experiments and manuscript preparation. ML assisted with protein purification, maintained cancer cell cultures and performed real-time qRT-PCR. All authors have read and approved the final manuscript.
